# Team-Based Learning for Immunology Courses in Allied Health Programs

**DOI:** 10.3389/fimmu.2019.02477

**Published:** 2019-10-30

**Authors:** Stephanie James, Peter Cogan, Marianne McCollum

**Affiliations:** Regis University School of Pharmacy, Denver, CO, United States

**Keywords:** Team-Based Learning, active learning, TBL, flipped class, pharmacy

## Abstract

Immunology is now a major component of studies in human biology, with many diseases having immune system involvement. Because so many areas of study include aspects of immunological knowledge, how to teach and incorporate immunology must be evaluated and assessed at all levels of education including K-12, undergraduate, graduate, medical, and professional programs. Traditional teaching methods such as lecture have significant shortcomings which make them less appealing to students today who are more digitally inclined and demand more active and engaging learning environments. Herein, we describe and propose the use of the active learning model of Team-Based Learning (TBL) to incorporate immunology into medical and professional programs. TBL is defined as an evidence based collaborative learning strategy taught in a three-step cycle: pre-class preparation, in-class readiness assurance testing (RAT), and application-focused exercises. In TBL, students are assigned to 6–7 member teams. Students complete the in-class RAT individually followed by taking the RAT as a team (T-RAT). Following the RAT and T-RAT, the instructor can then provide immediate feedback on concepts that proved especially difficult. The remainder of class time is then spent with teams working case studies and applications relative to the instructional topic or disease. Teams decide the best outcome or answer for a given application and report their answers simultaneously in class, followed by a discussion facilitated by the instructor. Research indicates that students involved in active learning classes, such as those using TBL have significantly increased levels of student engagement and high performance on examinations. This review will highlight how to implement TBL into a professional program (medical, dental, nursing, or pharmacy), how to assess student performance and provide real world examples of case studies and applications.

## Introduction

Collectively, the biological sciences have a general reputation of being difficult academic subjects. The reasons for this are varied and may include the “language of immunology” which has new key words (e.g., Cytokines, complement, various types of immune specific cells) that are not discussed in other biological fields.

The immune system can be characterized as an expansive network of tissues, cells, cytokines, and signaling processes which support the function of all other body systems. Consequently, failings of the immune system can have far reaching effects throughout the body. For example, autoimmune diseases such as Multiple Sclerosis present with neurological symptoms and patients are treated by experts in neurology. But it cannot be ignored that MS is an aberration of immune regulation involving T and B cells, among others. The transition of macrophages into foam cells during the development of heart failure is another example of where an aspect of the immune system overlaps with another disease state and body system. More obvious examples include the role of the immune system in fighting off various types of infectious diseases and cancers, as well as in its governance of immunization and organ transplantation outcomes.

Because of its foundational role in human health and pertinence across an array of disease states, it is imperative that students of the health sciences develop a strong appreciation for immunology and a robust understanding of immune function. A key question is how to best deliver immunology education. It has been well-documented that lecturing is a passive, and perhaps outdated, educational pedagogy. Students in lecture courses often report boredom and loss of interest after approximately 15 min of lecture. This means that for a 60-min class, only 25% of the material is truly delivered to the student. Furthermore, the delivery of material via lecture can often limit question and answer opportunities and decrease student engagement. Thus, in this review we propose Team-Based Learning (TBL) as a method to deliver immunology material with a particular emphasis on optimizing health professions education.

## Active Learning Using TBL

Active learning techniques have been celebrated for decades as promising solutions to the commonly perceived problems of student engagement and subject matter retention. Multiple reviews have covered the evidence base for the use of such paradigms in STEM (Science, Technology, Engineering, and Mathematics) education, with a focus on those methodologies which incorporate cooperative student groups engaged in problem solving exercises that require some degree of mastery of the pertinent subject matter (1–3). Such pedagogies can be described as *constructivist* in that they champion the notion of learners building knowledge for themselves, an approach that stands in stark contrast to the classic lecture model in which information is transmitted passively to students with expectations of memorization and little hope of integration (4). These constructivist strategies have been demonstrated, with varying success and significance, to improve student engagement, critical thinking, exam scores, pass rates, and retention rates in a variety of settings (3).

In 1992, Larry Michaelsen published the description of a novel approach to small group teaching which was intended to capitalize upon the strengths and address the shortcomings of other active learning strategies (5). This highly structured and intentional approach was ultimately branded as “Team-Based Learning” (TBL) and has subsequently been successfully employed across multiple STEM fields. More particularly, TBL is exquisitely suited to education in the allied health professions as it allows for an efficient treatment of meaningful, multivariate, and complex clinical situations through peer guided case assessment and active problem solving (6). This was demonstrated in a study by Burgess et al. (6) in which TBL was compared to Problem-based learning (PBL) in a cohort of medical students. Students utilized both PBL and TBL methodology to study musculoskeletal, cardiovascular, and respiratory units. At the end of the term students completed questionnaires regarding the strengths and weaknesses of each method in relation to their learning. Students favored the TBL format over the PBL and reported the decreased group size, pre-reading assignments, and assessment activities contributed to improved learning and better understanding of the material. Students also noted the immediate feedback from experts and relevant applications led to better engagement with the material and understanding.

In accordance with several recognized critical attributes of effective learning methods, TBL is a constructivist process which focuses on the acquisition of procedural knowledge and capitalizes on the ability of groups to learn more efficiently than individuals while relying on student ability to articulate explanations and defend group reasoning as part of the assessment of subject mastery (2). Haidet et al. have carried out an exhaustive review of the TBL literature and found that students in TBL courses reported (on average) higher levels of engagement and satisfaction (7). Additionally, those that were initially on the bottom of the curve reported improved individual outcomes. Furthermore, the TBL paradigm allows for the traverse of multiple tiers of Bloom's taxonomy via a three-phase process which capitalizes on peer interactions to build upon, and ultimately result in, the individual's competency and personal responsibility for learning (8). The example at the end of this review is a typical application that requires students to have read and learned basic immunological terms and concepts such as the various types of immune cells and their function, the process of inflammation and the role of some specific cytokines and pattern recognition receptors. TBL has successfully been employed as a pedagogy for the delivery of immunology course materials and has likewise enjoyed broad application and generated positive outcomes in a variety of related fields in both the basic and clinical sciences (9–13). Our purpose here is to present an example of a TBL application and assessments specific to the immunology content of coursework in the allied health professions.

## Structure and Student Assessment in TBL Classes

**Figure 1:**
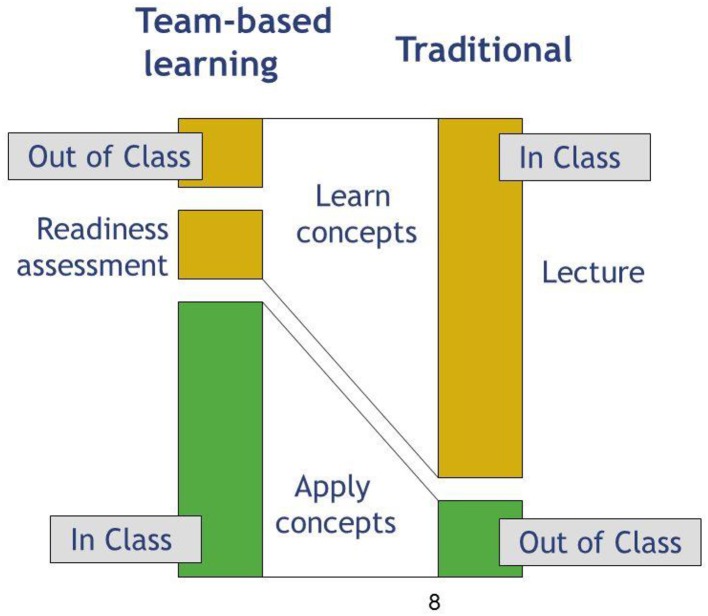
TBL utilizes a flipped classroom model, in which students are responsible for reading and reviewing material prior to class time, so the majority of class time is spent practicing applications and case studies with instructor guidance. This is in comparison to traditional lecture methods in which students would be introduced to material during a lecture.

Knowledge and basic comprehension are developed in phase one of the TBL process through individual preparation exercises which commonly take the form of pre-class readings guided by a list of specific learning objectives. This serves to enhance classroom efficiency by placing the onus of achieving lower levels of Bloom's Taxonomy on the learner (i.e., self-directed learning). That is, no class time need be spent on basic concepts, definitions, or terminology which the learner is expected to read and review ahead of time (Figure 1). In the example used at the end of this review, students will have read a primary literature article and been given supplemental information (in the form of textbook chapters, videos) on basic concepts in innate immunity. Students will also have completed a required microbiology course.

In phase two, individual and team readiness assurance tests (iRATs and tRATs, respectively) not only incentivize the completion of pre-class preparations, but also serve to clarify concepts and reinforce comprehension (14). After each student completes the 10–20 question iRAT on their own, they immediately reconvene with their team (4–6 other students) to work on the same assessment as a group. The tRAT is scored using a scratch-off card (Immediate Feedback Assessment Technique®, or IF-AT, card). IF-AT cards are similar to lottery tickets in that each team responds to tRAT items by scratching off their agreed-upon answer to reveal a star if they have chosen correctly. If an incorrect answer has been chosen, the team continues discussion and scratches off their second choice, repeating the process until the correct answer is identified. Full or partial credit (4 points, 2 points, 1 point, 0 points) is awarded based on the number of attempts needed to answer correctly.

The tRAT with IF-AT scoring supports learning in multiple ways and renders a superior feedback mechanism when compared to traditional assessments which require that students actively check numerical grades after-the-fact to determine what their knowledge deficits are. IF-AT cards provide immediate feedback to confirm knowledge and build student confidence while eliciting germane questions and correcting errors in thinking through team discussion in real time. Furthermore, the promise of partial credit serves to stimulate these continued discussions and fosters participation in iterative rounds of peer-to-peer teaching which reinforce and improve student understanding of material. In addition to these benefits associated with the immediate feedback afforded by IF-AT cards, the iRAT process can be further capitalized upon when coupled with a computer-based testing program which gives faculty immediate access to psychometric data and item performance. Such reports serve to identify items that are still unclear to a substantial number of students, thus affording the instructor the opportunity to address specific areas of confusion via moderated class discussion or mini-lecture.

**Figure 2:**
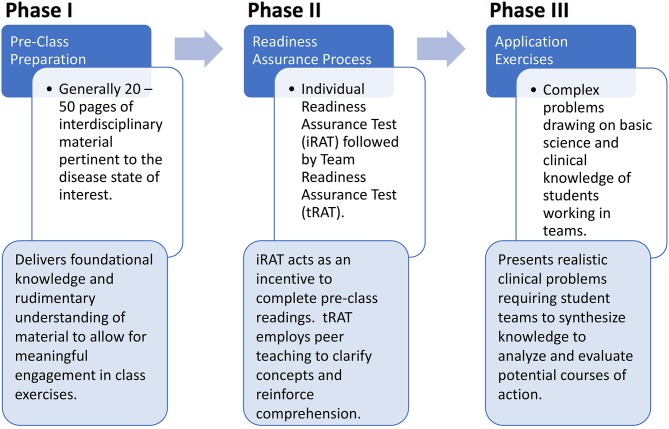
TBL uses 3 distinct phases: Prior to class students spend time independently reading and reviewing material assigned by the instructor. This is followed by completing a readiness assessment process (typically a quiz) to insure they are prepared for class. The final phase is completed during class time, and involves using the knowledge gained during phase I and II to evaluate and draw conclusions on practical applications or case studies.

After RATs are complete and knowledge deficits are addressed, the majority of class time can be spent on phase three, in which more complicated case studies and often confusing concepts can be covered through significant Application Exercises (AEs) aimed at achieving mastery of higher levels of Bloom's Taxonomy. AEs are solved at the team level and allow students to work together to apply the basic concepts learned in phases one and two. Faculty facilitation of AEs occurs at the class level and involves oral reports which require individuals to articulate and defend their team's rationale for answer selections/non-selections. Team-to-team discussions often ensue with instructor oversight, providing a rich environment for rigorous study of course material. As indicated in the application example, the instructor can use the questions to further engage students in a conversation regarding immunological processes and topics. The AE process is designed with the ultimate goal of empowering students to apply their knowledge toward the analysis and evaluation of potential courses of action in the context of realistic clinical problems. Multiple examples exist in the literature highlighting not only the ability of TBL to achieve these outcomes, but its further value in promoting professionalism, improved communication skills, and teamwork (15–17) (Figure 2).

To further encourage active engagement in class activities, students have the opportunity to assess their team-members in a round-robin peer evaluation process that covers a wide range of topics including inter-professional communication, contributions during class, and professionalism. Finally, an overarching assessment of student learning is achieved through individual midterm and final exams. Course grades are calculated as a weighted composite of individual performance (iRATs and examinations), team performance (tRATs and AEs), and peer evaluation scores. It is worth noting that the weighting of individual and team grade components deserves thoughtful treatment in order to avoid undesired outcomes such as excessive grade inflation, loafing, or individual students being “saved” by team performance, as team grades tend to be substantially higher than those achieved by individuals. One useful approach to guarding against the progression of dubiously qualified students is to base progression solely on the individual performance grade such that no student can progress without demonstrating competence. Using such a model, a threshold grade (e.g., 70%) must be achieved on exams, iRATs, and/or the combination of the two in order to pass the course, with team points being awarded to calculate the final grade once the pass/fail criteria has been met.

## Challenges Associated With TBL

TBL presents a number of challenges for both students and faculty. Students have typically spent many years learning passively with transfer of knowledge from faculty to student in lecture settings. TBL requires active engagement on the part of students as they take responsibility as life-long learners. The conversion from passive to active learning can take time and requires programmatic support of students making the transition. We have previously presented at the American Society of Health Pharmacists that students in a TBL program demonstrate improved problem-solving and critical thinking skills as well as improved study behaviors characterized by less cramming. In addition, students cite increased respect for the value of teams and the opinions of other team members along with an increased likelihood to share opinions with other team members, similar to observations reported by Luong et al. (18).

Challenges for faculty include pre-class preparation. In addition to researching, gathering, and writing pre-class preparation materials, RATs must be created and application exercises crafted to be of sufficient difficulty that they require a higher level of thinking on the part of the student. One way to accomplish this, particularly relevant for the health professions, is to utilize case studies. De-identified patient cases drawn from the personal experience of the facilitator often make for ideal AEs, though examples drawn from the literature can be effectively developed by faculty with minimal clinical experience and there are ample opportunities to deliver non-clinical basic science content through TBL (see example below). Finally, facilitation of TBL RATs and AEs requires faculty development in areas distinct from the ability to provide lectures. Faculty may initially be challenged in a TBL environment where student participation may often direct the course of discussions to elicit unforeseen questions and tangential explorations.

## An Example TBL Application

The application below is an example of how a study from the literature may be adapted to a TBL application exercise in an introductory pharmaceutical science class. This class topic was urinary tract infections, and students had previously studied sexually transmitted infections. Prior to class, students were required to read “Overdiagnosis of Urinary Tract Infection and Underdiagnosis of Sexually Transmitted Infection in Adult Women Presenting to an Emergency Department” by ME Tomas, which was published in *J Clin Microbiol* in 2015. This paper was a nice example to turn into an application as it is relevant to the class topic, provides engaging and relevant information for future health professionals, and gives the instructor the opportunity ask higher level Bloom's taxonomy questions regarding how the immune system responds to infections. The class was then given application questions:

1. Which immune cell would first respond to a UTI?
  - Macrophage
  - Neutrophil
  - Eosinophil
  - NK cell
  *The correct answer is B, neutrophils. Upon team reporting the instructor can use this opportunity to probe further as to how neutrophils are recruited to the site of an infection, what their role is in innate immunity*.
2. Would you expect the same cell type chosen in question 1 to be the first line defense in an infection with Herpes Simplex Virus-2?
  - Yes
  - No
  *The correct answer would be no. This question is more open ended and provides the instructor to discuss differences in immune responses to bacteria vs. viruses*.
3. Which of the following plays the MOST important role in the innate response in bladder epithelial cells upon initial infection?
  - TLR4
  - cAMP
  - TRPML3
  - Caspase-8
  *The correct answer should be A. This question helps the students understand the process of intracellular signaling and pattern recognition receptors such as TLR4 and the cascade of events which follows their engagement*.
4. A common STI is human papilloma virus, the most common cause of a UTI is *E. coli*. Since it is important not to delay therapy in either case, could you treat both of these bacteria with the same antibiotic?
  - Yes
  - No
  *The correct answer is no, because we do not treat viruses with an antibiotic. This question provides the instructor a nice opportunity to query students in differences of immune responses to viruses or bacteria*.

Following each question the facilitator discusses common symptoms and pathology, differences in presentation of different types of UTIs and importance of treatment. The third question also gives an opportunity to discuss differences in the types of bacteria that cause each disease, the mechanism of action of various antibiotic which may be used to treat.

## Conclusions

As our breadth of immunological knowledge expands, our approaches to education must also change. As mentioned, lecturing is now recognized as a passive form of education for the student that is not as effective at developing critical thinking skills as newer, active learning methods. As the development of active learning styles are evolving, TBL has emerged as an evidence based methodology that fosters improved critical thinking, better retention, and also has the advantage of developing “soft skills” among students including listening and communication skills. Such skills are particularly relevant for the health care professions, where providers must not only be experts in their field but must also be able to communicate effectively to their patients.

## Author Contributions

SJ conceived the idea for this paper, wrote, and edited. PC and MM wrote a significant part of this paper.

### Conflict of Interest

The authors declare that the research was conducted in the absence of any commercial or financial relationships that could be construed as a potential conflict of interest.
